# Genome-wide association study identifying novel risk variants associated with glycaemic traits in the continental African AWI-Gen cohort

**DOI:** 10.1007/s00125-025-06395-6

**Published:** 2025-03-01

**Authors:** Vivien J. Chebii, Alisha N. Wade, Nigel J. Crowther, Engelbert A. Nonterah, Godfred Agongo, Z. Simayi, Palwende R. Boua, Isaac Kisiangani, Michèle Ramsay, Ananyo Choudhury, Dhriti Sengupta, as members of the H3Africa AWI-Gen study

**Affiliations:** 1 Sydney Brenner Institute for Molecular Bioscience, Faculty of Health Sciences, University of the Witwatersrand, Johannesburg, South Africa; 2 Department of Internal Medicine, School of Clinical Medicine, University of the Witwatersrand, Johannesburg, South Africa; 3 Research in Metabolism and Endocrinology, Division of Endocrinology, Diabetes and Metabolism, Perelman School of Medicine, University of Pennsylvania, Philadelphia, PA USA; 4 MRC/Wits Rural Public Health and Health Transitions Research Unit, School of Public Health, Faculty of Health Sciences, University of the Witwatersrand, Johannesburg, South Africa; 5 Department of Chemical Pathology, National Health Laboratory Service, Faculty of Health Sciences, University of the Witwatersrand, Johannesburg, South Africa; 6 Navrongo Health Research Centre, Ghana Health Service, Navrongo, Ghana; 7 Department of Epidemiology, School of Public Health, C.K. Tedam University of Technology and Allied Sciences, Navrongo, Ghana; 8 Julius Global Health, Julius Centre for Health Sciences and Primary Care, University Medical Centre Utrecht, Utrecht University, Utrecht, the Netherlands; 9 Department of Biochemistry and Forensic Sciences, School of Chemical and Biochemical Sciences, C.K. Tedam University of Technology and Applied Sciences, Navrongo, Ghana; 10 Department of Pathology, Faculty of Health Sciences, University of Limpopo, Polokwane, South Africa; 11 Clinical Research Unit of Nanoro, Institut de Recherche en Sciences de la Santè, Nanoro, Burkina Faso; 12 MRC Unit The Gambia at London School of Hygiene and Tropical Medicine, Banjul, The Gambia; 13 African Population and Health Research Center, Nairobi, Kenya; 14 Division of Human Genetics, National Health Laboratory Service and School of Pathology, Faculty of Health Sciences, University of the Witwatersrand, Johannesburg, South Africa

**Keywords:** African ancestry, Fasting glucose, Fasting insulin, GWAS, Type 2 diabetes

## Abstract

**Aims/hypothesis:**

Glycaemic traits such as high fasting glucose levels and insulin resistance are positively associated with the risk of type 2 diabetes and other cardiometabolic diseases. Genetic association studies have identified hundreds of associations for each glycaemic trait, yet very few studies have involved continental African populations. We report the results of genome-wide association studies (GWASs) in a pan-African cohort for four glycaemic traits, namely fasting glucose, fasting insulin, insulin resistance (HOMA-IR) and beta cell function (HOMA-B), which are quantitative variables that affect the risk of developing type 2 diabetes.

**Methods:**

GWASs for the four traits were conducted in approximately 10,000 individuals from the Africa Wits-INDEPTH Partnership for Genomics Studies (AWI-Gen) cohort, with participants from Burkina Faso, Ghana, Kenya and South Africa. Association testing was performed using linear mixed models implemented in BOLT-LMM, with age, sex, BMI and principal components as covariates. Replication, fine mapping and functional annotation were performed using standard approaches.

**Results:**

We identified a novel signal (rs574173815) in the intron of the ankyrin repeat domain 33B (*ANKRD33B*) gene associated with fasting glucose, and a novel signal (rs114029796) in the intronic region of the WD repeat domain 7 (*WDR7*) gene associated with fasting insulin. SNPs in *WDR7* have been shown to be associated with type 2 diabetes. A variant (rs74806991) in the intron of ADAM metallopeptidase with thrombospondin type 1 motif 16 (*ADAMTS16*) and another variant (rs6506934) in the β-1,4-galactosyltransferase 6 gene (*B4GALT6*) are associated with HOMA-IR. Both *ADAMTS16* and *B4GALT6* are implicated in the development of type 2 diabetes. In addition, our study replicated several well-established fasting glucose signals in the *GCK-YTK6*, *SLC2A2* and *THORLNC* gene regions.

**Conclusions/interpretation:**

Our findings highlight the importance of performing GWASs for glycaemic traits in under-represented populations, especially continental African populations, to discover novel associated variants and broaden our knowledge of the genetic aetiology of glycaemic traits. The limited replication of well-known signals in this study hints at the possibility of a unique genetic architecture of these traits in African populations.

**Data availability:**

The dataset used in this study is available in the European Genome–Phenome Archive (EGA) database (https://ega-archive.org/) under study accession code EGAS00001002482. The phenotype dataset accession code is EGAD00001006425 and the genotype dataset accession code is EGAD00010001996. The availability of these datasets is subject to controlled access by the Data and Biospecimen Access Committee of the H3Africa Consortium. GWAS summary statistics are accessible through the NHGRI-EBI GWAS Catalog (https://www.ebi.ac.uk/gwas/).

**Graphical Abstract:**

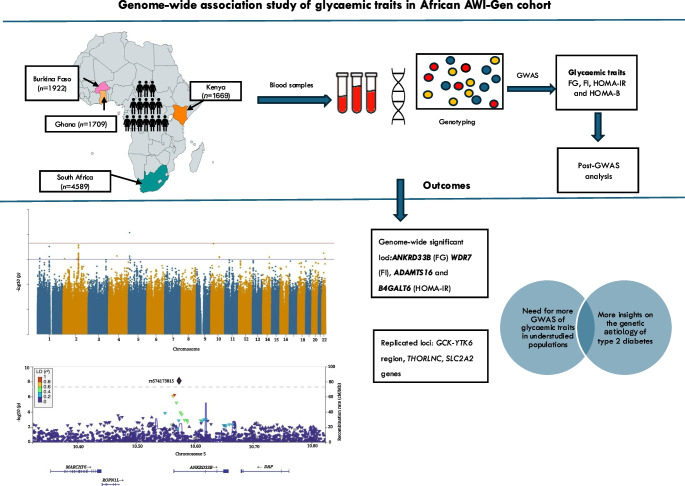

**Supplementary Information:**

The online version of this article (10.1007/s00125-025-06395-6) contains peer-reviewed but unedited supplementary material.


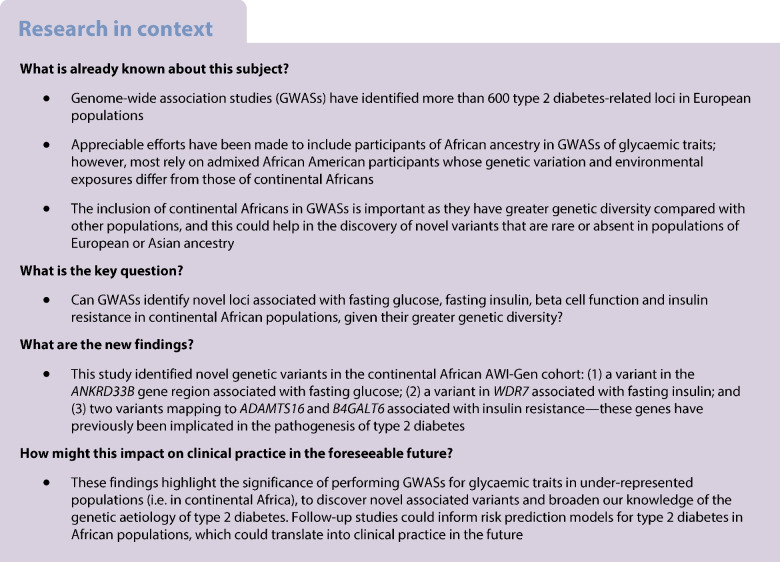


## Introduction

The disease burden for diabetes mellitus is significant, with approximately 24 million affected individuals residing in Africa in 2021 [1]. The prevalence of diabetes has been increasing in sub-Saharan Africa, with 55 million individuals in the subcontinent expected to have the disease by 2045 [1]. Type 2 diabetes is a complex disease characterised by elevated blood glucose levels due to insulin resistance and beta cell dysfunction [2, 3]. Abnormal values for several glycaemic traits, including fasting glucose (FG), 2 h glucose, fasting insulin (FI), HbA_1c_, HOMA-B and HOMA-IR, are often observed before clinical diagnosis of type 2 diabetes.

The discovery of genetic factors underlying glycaemic traits is crucial for understanding the aetiology of type 2 diabetes. Genetic studies aimed at identifying associations of variants with variables related to the risk for diabetes have predominantly included non-African cohorts [4]. Despite a distinct genetic make-up characterised by higher genetic diversity and shorter linkage disequilibrium blocks, the genetic architecture of glycaemic traits in continental Africans is under-studied [5]. Efforts have been made to include individuals of African ancestry in genome-wide association studies (GWASs), but these mainly using admixed African American participants. African Americans represent a small portion of Africa’s genetic diversity, and their environment, diet and lifestyles differ from those of populations in Africa [6, 7]. The inclusion of continental Africans in GWASs is pertinent because of their high disease risk [8]. A handful of GWASs using datasets from continental African populations have been conducted, including studies by Rotimi et al [9] and Chen et al [10]. However, both studies comprised case–control GWASs of type 2 diabetes, and did not investigate the quantitative variables related to risk for type 2 diabetes. There is only a partial overlap between glycaemic trait loci and type 2 diabetes loci [11], so genomic analysis of FG, FI, HOMA-B and HOMA-IR as quantitative variables is needed to complement the genetic analysis of type 2 diabetes as a dichotomous trait [12]. To our knowledge, the study by Gurdasani et al [13] is the only study that explored quantitative glycaemic traits in continental African populations. However, they only performed the GWAS for HbA_1c_.

In this study we performed a GWAS for FG, FI, HOMA-B and HOMA-IR using datasets drawn from African participants in the Africa Wits-INDEPTH Partnership for Genomics Studies (AWI-Gen) cohort. AWI-Gen is a population study of 12,000 older adults from four sub-Saharan African countries: Ghana and Burkina Faso in West Africa, Kenya in East Africa, and South Africa [14].

## Methods

### Ethics

The Human Research Ethics Committee (Medical) of the University of Witwatersrand, South Africa, approved the AWI-Gen study (renewal ethics approval code M2210108). Additional ethics approvals were obtained from the AWI-Gen study sites in Nanoro (Burkina Faso), Navrongo (Ghana), Nairobi (Kenya) and Limpopo (South Africa). The participants consented in writing before being recruited into the study.

### Study cohort

Participants were from AWI-Gen, a collaborative centre under the Human Heredity and Health in Africa Consortium (H3A), which aims to identify genetic and environmental factors that contribute to cardiometabolic disorders in African populations [15]. The AWI-Gen cohort includes 12,000 participants aged 40–60 years from East, West and Southern Africa, excluding pregnant women, recent immigrants, first-degree relatives of existing participants and individuals with physical impairments. Additional information on the inclusion and exclusion criteria has been published previously [14]. Participants with known diabetes were excluded from this study: an individual was considered diabetic if they had been previously diagnosed by a health professional, were receiving diabetes treatment, or had a fasting plasma glucose ≥ 7 mmol/l or random plasma glucose ≥ 11.1 mmol/l [8]). Characteristics of the study participants are provided in electronic supplementary material (ESM) Table 1.

### Glycaemic trait measurements

A detailed description of the data and sample collection methods has been presented by Ali et al [14]. Briefly, fasting blood samples were obtained from study participants after an overnight fast. Fasting plasma glucose was assayed with a Randox Plus clinical chemistry analyser using a colorimetric method. A solid-phase, enzyme-labelled chemiluminescent immunometric assay (Immulite 1000 chemistry analysis system, Siemens) was used to determine fasting serum insulin concentrations. HOMA-IR and HOMA-B were calculated as described previously [16, 17]. The ESM Methods provides more details on glycaemic measurements.

### Genotyping, quality control analysis and imputation

DNA samples from approximately 11,000 participants were genotyped on the 2.3 million H3Africa SNP array. Genotype calling was performed using an Illumina pipeline, and pre-imputation quality control analysis was performed using the H3ABioNet/H3Agwas pipeline (https://github.com/h3abionet/h3agwas) [18]. Briefly, samples with missing SNP calling rates greater than 0.05, discordant sex information and potential duplicates (pairwise identity by descent heterozygosity-adjusted trait [PIHAT] >0.9) were excluded. Similarly, individuals with heterozygosity <0.15 or >0.343 were removed. Additionally, SNPs with genotype missingness greater than 0.05, a minor allele frequency (MAF) less than 0.01, and those that showed extreme deviation from Hardy–Weinberg equilibrium (*p*<0.0001) were excluded. Non-autosomal chromosomes or mitochondrial SNPs, and SNPs that did not correspond to Human Build 37 (GRCh37) reference alleles or strands, were also excluded. A flow diagram showing the quality control steps is provided in ESM Fig. 1. Principal components were estimated using EIGENSTRAT [19]. Based on an in-depth evaluation [20], it was found that eight principal components were sufficient to address the population structure in our dataset.

Imputation was performed on the cleaned dataset using the African Genome Resources reference panel of the Sanger imputation server (https://imputation.sanger.ac.uk/). Further quality control analysis was performed after imputation: poorly imputed SNPs (info score ≤0.6) and SNPs with MAF ≤0.01 were removed. The final quality-controlled imputed dataset had 13.98 million SNPs, and only individuals with a phenotype meeting the inclusion criteria (see Study cohort section) for FG (*n*=9889), FI (*n*=6825), HOMA-B (*n*=6362) and HOMA-IR (*n*=6500) were used for association analyses.

Power calculations were performed using the gwas_power function in R software, version 4.2.1 [21] (see ESM Methods). The SNP heritability and genetic correlations of the four glycaemic traits were determined using GCTA-GREML, version 1.93.253 [22] (see ESM Methods). Cross-ancestry genetic correlation was estimated using POPCORN [23] (see ESM Methods).

### Genome-wide association analysis

Genome-wide association analyses were performed using the linear mixed model implemented in BOLT-LMM software, version 2.3.4 [24] for FG, FI, HOMA-B and HOMA-IR. Untransformed FG, log-transformed FI, log-transformed HOMA-B and log-transformed HOMA-IR were used, with sex, BMI, age and eight principal components as covariates. GWASs were also performed on regional subsets of the data (West African, East African and South African) using REGENIE, version 3.4.1 [25]. As there is no consensus on the ideal *p* value threshold for African GWASs, we employed the commonly used genome-wide significance criterion of *p*<5 × 10^−8^ [20]. Any SNPs with a *p* value between 5 × 10^−8^ and 1 × 10^−6^ were considered to be suggestive. Manhattan and quantile–quantile (QQ) plots were generated from the GWAS results using the qqman package in R version 4.2.0, together with estimation of the genomic control inflation factor (λ) [26].

### Functional analysis of associated variants and fine mapping

The genome-wide significant variants were annotated using the Ensembl variant effect predictor [27]. The function of mapped genes was inferred using GeneCards [28]. Annotation and prioritisation of genomic risk loci were performed using the Functional Mapping and Annotation (FUMA) web tool [29] (see ESM Methods). The significantly associated loci were cross-referenced with the GWAS Catalog [30] to identify previously reported signals.

The program FINEMAP [31], implemented in the H3Agwas pipeline [18], was used to find candidate causal variants. The full AWI-Gen genotype data and site-specific genotype data were used as linkage disequilibrium (LD) references for fine mapping. FINEMAP employs a Bayesian-based framework to predict the posterior probabilities (*pp*) of causative variants using summary statistics and LD correlations among variants [31] (see ESM Methods). Independently associated SNPs were selected using a stepwise model selection procedure in COJO-GCTA [32], using AWI-Gen genotype data as the LD reference data.

### Assessment of transferability of previously detected signals to the AWI-Gen dataset

Replications of known FG-, FI-, HOMA-IR- and HOMA-B-associated SNPs reported in the GWAS Catalog [30] and other sources [7, 33–37] were performed using custom scripts. The GWAS Catalog data (https://www.ebi.ac.uk/gwas/, accessed on 15 Jan 2024) was converted from the human genome build GRCh38 to build GRCh37 using the liftOver tool [38] to allow comparison with the results in our study. Keywords relevant to glycaemic traits (‘fasting blood glucose’, ‘blood glucose’, ‘fasting blood insulin’ and ‘insulin’) were used to identify associations from the GWAS Catalog dataset. Only SNPs with the same risk allele and direction of effect as SNPs reported in our cohort were considered transferable. The transferability of genetic associations was assessed at a relaxed replication threshold of *p*<0.005, as previously suggested [39, 40]. None of the GWAS loci for HOMA-B and HOMA-IR in the MAGIC consortium were significant; hence, we performed replication using only data from the GWAS Catalog.

### Lookups of genome-wide signals

Lookups of the signals detected in this study were performed using trans-ancestral MAGIC datasets [7] and African American Glucose and Insulin Genetic Epidemiology (AAGILE) Consortium datasets [35]. The genomic build of the summary statistics for AAGILE [35] was converted to the human genome build GRCh37 using the liftOver tool [38]. Additionally, lookups were performed using data from type 2 diabetes loci from the African population [10] and HbA_1c_ loci from the Ugandan cohort [13].

## Results

### Study overview

The study was based on genotype and phenotype data from 9889 participants for FG, 6825 for FI, 6501 for HOMA-IR and 6360 for HOMA-B. The participants were aged 40–60 years, with a mean age (± SD) of 51.5 ± 7.9 years and a mean BMI (± SD) of 24.9 ± 6.4 kg/m^2^. The mean FG was 4.9 ± 0.9 mmol/l. The mean FI was 89.5 ± 151.9 pmol/l, the mean for HOMA-IR was 3.3 ± 5.7, and the mean for HOMA-B was 25 ± 105. Characteristics of the study participants are given in ESM Table 1.

The power estimates show that our GWASs for the four traits had sufficient power (>80%) to identify variants with an effect size greater than 0.27 and an MAF greater than 0.04 (see ESM Figs 2–5). The SNP-based heritability of the four glycaemic traits after adjusting for age, sex and principal components was as follows: for FG, h^2^=0.11, SE=0.04; for FI, h^2^=0.03, SE=0.04; for HOMA-IR, h^2^=0.06, SE=0.06; for HOMA-B, h^2^=0.07, SE=0.05. The genetic correlation (*r*_g_) between FG and FI was 0.20 (SE=0.18), that between FG and HOMA-IR was 0.47 (SE=0.15), that between FG and HOMA-B was −0.18 (SE=0.25) and that between FI and HOMA-IR was 0.96 (SE=0.02). Cross-ancestry genetic correlation results were not generated because the POPCORN method failed to converge for these traits.

### Genome-wide significant SNPs

We identified one genome-wide significant locus associated with FG and one associated with FI (Table 1). Two loci were identified as associated with HOMA-IR (Table 1). Although no genome-wide associations were detected for HOMA-B, SNPs in seven independent loci reached the suggestive significance threshold (*p*<1 × 10^−6^) (ESM Table 2). The region-wise GWAS results are provided in ESM Table 2.

**Table 1:** Genome-wide significantly associated SNPs (*p*<5 × 10^−8^) for glycaemic traits in the African AWI-Gen cohort

Trait	Nearest gene	SNP	Chromosome: bp	EA/NEA	EAF	β	SE	*p* value
FG	*ANKRD33B*	rs574173815	5: 10,573,505	T/C	0.015	0.304	0.052	7.4 × 10^−9^
FI	*WDR7*	rs114029796	18: 54,671,956	G/A	0.023	0.141	0.025	9.9 × 10^−9^
HOMA-IR	*ADAMTS16*	rs74806991	5: 185,200,578	T/G	0.050	0.104	0.018	2.1 × 10^−8^
HOMA-IR	*B4GALT6*	rs6506934	18: 29,215,618	G/A	0.250	0.048	0.009	2.8 × 10^−8^

The bp position is based on human genome build GRCh37

EA, effect allele; NEA, non-effect allele; EAF: effect allele frequency; β, effect size

### Associations for fasting glucose levels

**Fig. 1:**
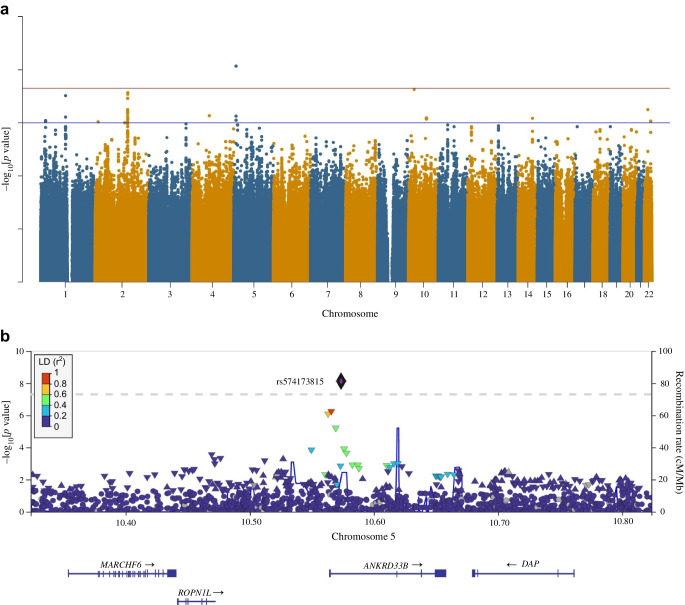
(**a**) Manhattan plot for the FG GWAS. The red horizontal line indicates the genome-wide significance threshold (*p*=5 × 10^−8^); the blue horizontal line shows the suggestive significance threshold of *p*=1 × 10^−6^. (**b**) Regional plot of chromosome 5 (including the *ANKRD33B* gene region), showing association with FG in the African AWI-Gen cohort: the lead SNP (rs574173815) is shown as a black and purple diamond, and SNPs in LD with the lead SNP are shown as coloured triangles. The *x*-axis shows the genomic positions based on the Human Build 37 reference genome (GRCh37). The blue peaks indicate the recombination rates at each position. Genes overlapping with or near the locus are shown below the *x*-axis. LD was based on the African 1000 Genomes Project reference panel

The Manhattan and QQ plots for FG associations are shown in Fig. 1a and ESM Fig. 6. The only genome-wide significant association (rs574173815, *p*=7.4 × 10^−9^, β=0.304) was localised to the intronic region of the *ANKRD33B* gene (Table 1, Fig. 1b). Although *ANKRD33B* has not been previously linked to any glycaemic traits, variants in this gene have been associated to obesity-related traits [41, 42]. Twenty-nine suggestive variants (*p*<1 × 10^−6^) associated with FG were reported in seven loci, including the intronic regions of *ZBTB40* (rs150293241 and rs563859139), *FRAS1* (rs77548352) and *SYN3* (rs57574537) (ESM Table 2).

Of the 24 FG variants reported in the MAGIC African American dataset, six SNPs in the *GCK-YTY6* region were replicated in our dataset (Table 2). Four of these SNPs (rs730497, rs2971670, rs1799884 and rs2908286) mapped to the intronic region of *GCK*, whereas the other two (rs2971667 and rs2908282) mapped to the intronic region of *YTK6*. Nine FG loci in the MAGIC European dataset (five after LD pruning) were replicated in the AWI-Gen cohort, with 98% of the variants showing consistent effect-size directions (ESM Table 3). Similarly, we replicated 12 FG loci in the European population in the UK Biobank (eight after LD pruning), with 98% also showing consistent effect-size directions [36] (ESM Table 4). Four additional loci in a multi-ethnic study [34] were also replicated; 94% of the variants showed consistent effect-size directions (Table 2; ESM Table 5). Notably, we replicated an FG locus on chromosome 7 (*GCK-YTY6* region) across multiple ancestry groups. Only two of 400 SNPs previously reported to be associated with FG in the GWAS Catalog were replicated in the AWI-Gen dataset. One signal was reported in the *GCK-YTK6* region in non-MAGIC cohorts in the GWAS Catalog, while the other signal was reported in the *THORLNC* gene region (Table 2) in East Asians [43].

**Table 2:** Genome-wide significant fasting glucose-associated loci reported in previous studies that attained our replication threshold (*p*<0.005) in the AWI-Gen cohort

Chromosome: bp	Gene region	SNP	EA/NEA	EAF	β	SE	*P*_AWI-Gen	R_ COHORT	R_ EAF	R_BETA	R_SE	R_*P*
2: 11,889,336	*THORLNC*	rs3849330	A/T	0.424	0.076	0.012	2.6 × 10^−3^	1	0.288	0.021	0.014	5 × 10^−9^
3: 170,726,363	*SLC2A2*	rs11711437	G/C	0.528	0.06	0.012	1.10 × 10^−6^	2	0.138	0.008	0.0003	5.41 × 10^−73^
3:170,727,351	*SLC2A2*	rs1879442	A/G	0.63	0.054	0.013	2.80 × 10^−5^	2	0.289	0.007	0.0004	7.64 × 10^−117^
3: 170,727,739	*SLC2A2*	rs5402	A/T	0.494	0.055	0.012	7.00 × 10^−6^	2	0.133	0.008	0.0004	9.56 × 10^−70^
3: 170,729,414	*SLC2A2*	rs61791106	A/G	0.494	0.056	0.012	4.40 × 10^−6^	2	0.136	0.008	0.0004	1.85 × 10^−72^
3: 170,729,873	*SLC2A2*	rs6785803	C/G	0.494	0.056	0.012	4.40 × 10^−6^	2	0.136	0.008	0.0005	2.41 × 10^−72^
3: 170,732,300	*SLC2A2*	rs5400	A/G	0.441	056	0.012	6.50 × 10^−6^	2	0.136	0.008	0.0003	4.86 × 10^−73^
3: 170,736,708	*SLC2A2*	rs11917504	T/A	0.559	0.056	0.012	6.60 × 10^−6^	2	0.128	0.007	0.0005	3.20 × 10^−59^
3: 170,740,071	*SLC2A2*	rs7646014	C/G	0.559	0.056	0.012	6.20 × 10^−6^	2	0.131	0.007	0.0004	1.88 × 10^−61^
7: 44,245,060	*YKT6*	rs2971667	C/T	0.347	0.039	0.013	3.3 × 10^−3^	3	0.039	0.008	0.008	2.825 × 10^−8^
7: 44,223,721	*GCK*	rs730497	A/G	0.220	0.063	0.016	3.5 × 10^−5^	4	0.191	0.055	0.009	6.435 × 10^−12^
								1	0.178	0.072	0.06	1 × 10^−32^
								2	0.177	0.013	0.0004	1.52 × 10^−275^
7: 44,226,101	*GCK*	rs2971670	T/C	0.234	0.059	0.015	7.8 × 10^−5^	3	0.193	0.061	0.009	1.102 × 10^−13^
								1	0.180	0.014	0.013	4 × 10^−277^
7: 44,229,068	*GCK*	rs1799884	T/C	0.213	0.066	0.015	1.6 × 10^−5^	3	0.193	0.061	0.009	1.285 × 10^−13^
								1	0.180	0.060	0.040	4 × 10^−28^
								4	0.112	0.047	0.007	2.023 × 10^−10^
								2	0.177	0.013	0.0004	2.04 × 10^−274^
7: 44,234,737	*GCK*	rs2908286	T/C	0.233	0.059	0.015	7.9 × 10^−5^	3	0.188	0.063	0.009	3.451 × 10^−14^
7: 44,248,828	*YKT6*	rs2908282	A/G	0.268	0.055	0.014	1.2 × 10^−4^	3	0.274	0.043	0.008	2.548 × 10^−9^
								4	0.192	0.039	0.007	1.986 × 10^−8^

The bp position is based on human genome build GRCh37

A subset of the replicated loci reported by Lagou et al [34] is presented in the table; the remainder are provided in ESM Table 5

EA, effect allele; NEA, non-effect allele, β, effect size; *P*_AWI-Gen, *p* value for the AWI-Gen cohort; R_cohort, replication cohort: where the numbers represent the dataset sources as follows: (1) GWAS Catalog [30]; (2) Lagou et al [34]; (3) MAGIC African American population [7]; (4) African Americans in the AAGILE study [35]; R_EAF, R_BETA, R_SE and R_*P* are the effect allele frequency, effect size, SE and *p* values for the replicated variants in each respective replication cohort

Lookup analysis showed that the lead variant for FG (rs574173815) reported in AWI-Gen cohort was absent in MAGIC datasets except in the European ones, where it showed no significant association (*p*=0.64). Fine mapping of the genomic region 1000 bp upstream and downstream of *ANKRD33B* showed that rs574173815 (*pp*=0.99) had a Bayes factor (log_10_bf) of 6.7; hence, it was the only candidate causal variant driving FG association (ESM Table 6). Conditional analysis did not yield any secondary signals associated with FG.

### Association with fasting insulin

**Fig. 2:**
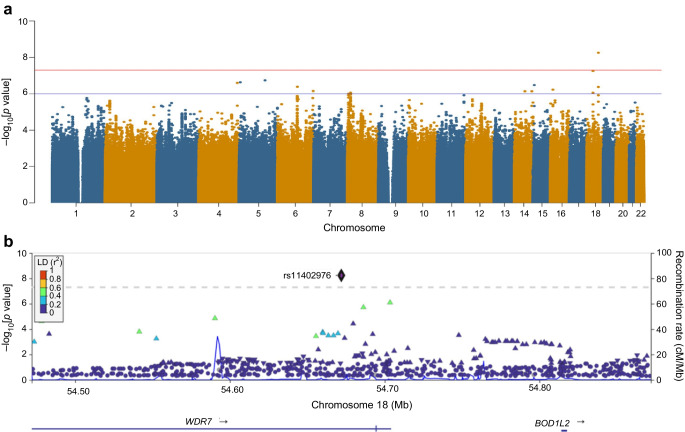
(**a**) Manhattan plot for the FI GWAS. The red horizontal line indicates the genome-wide significance threshold (*p*=5 × 10^−8^); the blue horizontal line shows the suggestive significance threshold of *p*=1 × 10^−6^. (**b**) Regional plot of chromosome 18 (*WDR7* and *BODIL2* gene region) showing associations with FI in the African AWI-Gen cohort: the lead SNP (rs114029796) is shown as a black and purple diamond, and SNPs in LD with the lead SNP are shown as coloured triangles. The *x*-axis shows the genomic positions based on the Human Build 37 reference genome (GRCh37). The blue peaks indicate the recombination rates at each position. Genes overlapping with or near the locus are shown below the *x*-axis. LD was based on the African 1000 Genomes Project LD panel

The Manhattan plot for the FI GWAS is shown in Fig. 2a. The corresponding QQ plot is shown in ESM Fig. 7. We identified one signal for FI (rs114029796, *p*=9.9 × 10^−9^) in the intronic region of *WDR7* gene and 142 kb upstream of the *BOD1L2* gene. Both *WDR7* and *BOD1L2* have previously been reported to be associated with type 2 diabetes [28, 44]. While the lead variant rs114029796 has not been linked to FI, a nearby variant (rs17684074), located 3 kbp upstream of it, has been previously associated with type 2 diabetes [44]. Similarly, another variant (rs10048404), 93 kbp downstream of SNP rs114029796, has been shown to be associated with type 2 diabetes [45]. Variants near *WDR7* that were previously reported to be associated with type 2 diabetes were tested in the AWI-Gen cohort but were not replicated. The pairwise LD between rs17684074 and rs11402979 was approximately 0.005, while the pairwise LD between rs10048404 and the lead SNP was approximately 0.0008 in the AWI-Gen cohort. We detected 13 variants for FI in six loci at the suggestive significance threshold (*p*<1 × 10^−6^) (ESM Table 2).

None of the associations for FI in the AAGILE dataset were significant; therefore, replication was not performed using this dataset. We did not replicate FI signals in the MAGIC African dataset (ESM Table 7), and only one FI locus from the GWAS Catalog was replicated (ESM Table 7). Additionally, we did not replicate FI signals using GWAS data relating to fasting proinsulin [33] at our predefined threshold of *p*<0.005. However, by applying a more relaxed threshold of *p*<0.05, we replicated four loci (16 variants) associated with FI in the fasting proinsulin study (ESM Table 7). No FI signals in a postprandial insulin resistance study were replicated [37]. Fine mapping of the *WDR7* locus showed that rs114029796 (*pp*=0.973; log_10_bf=6.5) was a likely candidate causal variant associated with FI. Conditional analysis did not yield any secondary signals associated with FI.

### Association with HOMA-IR

**Fig. 3:**
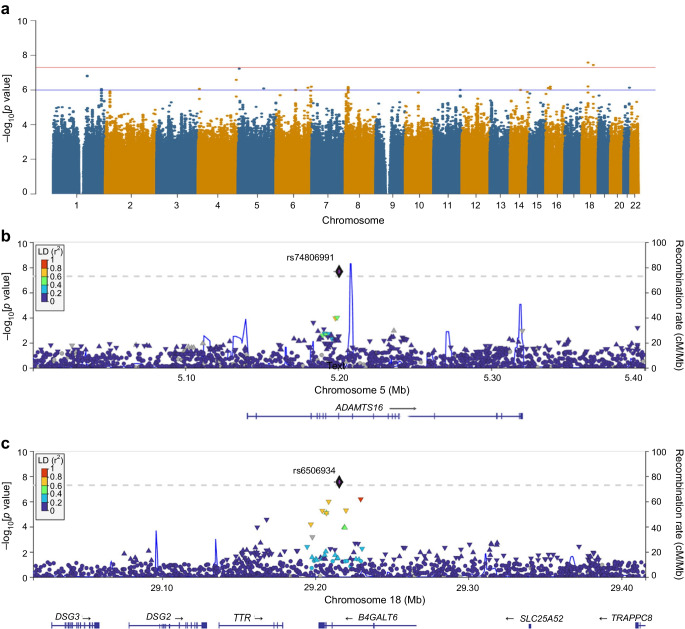
(**a**) Manhattan plot for the HOMA-IR GWAS. The red horizontal line indicates the genome-wide significance threshold (*p*=5 × 10^−8^); the blue horizontal line shows the suggestive significance threshold of *p*=1 × 10^−6^. (**b**) Regional plot of chromosome 5 (including the *ADAMTS16* gene region) showing association with HOMA-IR in the African AWI-Gen cohort: the lead SNP (rs74806991) is shown as a black and purple diamond, and SNPs in LD with the lead SNP are shown as coloured triangles. (**c**) Regional plot of chromosome 18 (including the *B4GALT6* gene region) showing an association with HOMA-IR in the African AWI-Gen cohort: the lead SNP (rs6506934) is shown as a black and purple diamond, and SNPs in LD with the lead SNP are shown as coloured triangles. The *x*-axis shows the genomic positions based on the Human Build 37 reference genome (GRCh37). The blue peaks indicate the recombination rates at each position. Genes overlapping with or near the locus are shown below the *x*-axis. LD was based on the African 1000 Genomes Project LD panel

The HOMA-IR associations are summarised in Fig. 3a. The QQ plot confirms the absence of any inflation (λ=0.998) (ESM Fig. 8). Two loci (each with a single variant) associated with HOMA-IR reached genome-wide significance (rs74806991, *p*=2.1 × 10^−8^ and rs6506934, *p*=2.8 × 10^−8^). The variant rs74806991 mapped to the coding region of *ADAMTS16*, while rs6506934 mapped to *B4GALT6*. Several signals associated with FI also reached the suggestive significance threshold in the HOMA-IR GWAS (ESM Table 2), underscoring the overlap between the genetic architecture of these two related traits. None of HOMA-IR-associated SNPs reported previously were replicated in our dataset (ESM Table 7). Fine mapping showed that lead SNPs at both *B4GALT6* (rs6506934; *pp*=0.89) and *ADAMTS16* (rs74806991; *pp*=0.99) are candidate causal variants associated with insulin resistance.

## Discussion

In this study, we harnessed phenotype and genotype data from sub-Saharan African populations to explore the genetic basis of four glycaemic traits: FG, FI, insulin resistance and beta cell function. We identified one signal (*ANKRD33B*, rs574173815) associated with FG. The effect allele (rs574173815-T) of the variant associated with FG has a modest frequency (2.7%) in the African population of the 1000 Genomes Project, and a frequency of 1.5% in the AWI-Gen cohort, but was absent in all other population groups in the 1000 Genomes Project with the exception of the American population, where it was present at a low frequency (0.1%) [46]. This variant was absent in global GWAS results, and may be specific to continental Africans, and this explains why we did not replicate it. The biological role of *ANKRD33B* in association with FG remains unclear. The report of an independent association (rs4702718-G) in this gene with energy intake [42] suggests a possible connection to glucose level and highlights the necessity for its further consideration.

The GWAS for FI identified one signal (rs114029796) in an intron of the *WDR7* gene, which was supported by several suggestive associations that are in high LD. The effect allele of SNP rs114029796 has a frequency of 2.3% in the AWI-Gen cohort, and is present at an allele frequency of 2% in the African population of the 1000 Genomes Project, but was absent in all other population groups in the 1000 Genomes Project except for the American population, where it was present at a low frequency (0.7%) [46]. Its low frequency and absence in major populations may explain the lack of replication of this signal. The lead variant in *WDR7* has not be reported to be associated with FI in GWASs; however, other variants in this locus are linked to type 2 diabetes [45].

Furthermore, we identified two loci – *ADAMTS16* (rs74806991) and *B4GALT6* (rs6506934) – associated with HOMA-IR. *ADAMTS16* encodes a metalloproteinase that belongs to the ADAMTS family [47]. *ADAMTS16* has not been associated with any glycaemic traits in GWASs; however, a SNP in *ADAMTS9* has been shown to be associated with increased type 2 diabetes risk, suggesting that *ADAMTS* gene family members contribute to diabetes pathophysiology [48]. The protein encoded by the second genome-wide significant locus (*B4GALT6*) catalyses the synthesis of ceramides and has been shown to be associated with type 2 diabetes [49]. rs74806991 and rs6506934 are promising variants of interest in insulin resistance and warrant further studies. Although no genome-wide significant signals were reported for HOMA-B, we expect that the GWAS data may make valuable contributions to future meta-analyses for this trait, especially in African populations.

The SNP-based heritability (h^2^SNP) estimate for FG was 11%, indicating a significant contribution of genetic variation, whereas the SNP heritability estimates for FI, HOMA-IR and HOMA-B were 3%, 6% and 7%, respectively, indicating moderate genetic contribution. We found positive genetic correlations between FG and FI, and FG and HOMA-IR, and negative correlations between FG and HOMA-B. The genetic correlation (*r*_g_) between FG and FI was weak (0.2), which may be attributed to the modest sample size. Similarly, we observed a positive correlation between FI and FG, FI and HOMA-IR, and FI and HOMA-B, with the highest correlation observed between FI and HOMA-IR (*r*_g_=0.96, SE=0.02), as reported previously [50].

Several SNPs in the *GCK-YTK6* and *SLC2A2* loci that were previously shown to be associated with glycaemic traits in various GWASs across various ancestries were replicated in our cohort at a relaxed replication significance threshold. While the overall low replication rate in this study may be attributed to the modest sample size and limited power to detect variants with a small effect size (<0.22) and MAF (<0.04) (ESM Fig. 1), a careful look at statistics from African-ancestry GWASs for diabetes-related traits suggests that this problem is not unique to our study. For instance, only 1% (60/6550) of FG signals detected in the European MAGIC set were replicated in the GWASs based only on African populations at a relaxed threshold of *p*<0.05 (corrected for multiple testing). The rate of replication for FI signals from European to African populations in the MAGIC study was 0.4% (9/2247) (*p*<0.05, corrected for multiple testing). If we apply the same *p* threshold used in our cohort to the study by Adeyemo et al [51], only 5% (6/108) of type 2 diabetes associations tested were replicated, while the study by Chen et al [10] replicated 4% (4/100) of type 2 diabetes signals detected in non-African cohorts in continental Africans, which is comparable to our results. Although the study by Gurdasani et al [13] replicated 52% (344/658) of HbA_1c_ signals in the MAGIC African American dataset, they were all in sex chromosomes.

Our previous work on lipid traits conducted on the same cohort was able to replicate almost all the strong signals from the PAGE study by Choudhury et al [20], implying that the lack of replication may not only be due to technical aspects such as sample size and power, but may also hint at underlying biological differences. It is important to note that the pathogenesis for type 2 diabetes and related glycaemic traits has been reported to differ by ethnicity, especially between African and non-African populations [52]. It has been suggested that, for African populations, low insulin sensitivity and hyperinsulinaemia (due to reduced hepatic clearance) may be the primary defect, which is not the case for non-African populations [53]. Therefore, large-scale African studies are needed to assess whether the differences in pathophysiology of type 2 diabetes (and related quantitative traits such as FG and FI) is related to differences in the genetic architecture of these traits.

The choice of *p* value threshold for genome-wide significance as well as for replication has a strong impact on the results presented. There are valid concerns about whether the standard and widely used GWAS significance *p* threshold of 5 × 10^−8^ needs to be revised, as GWASs conducted on imputed datasets and sequence datasets perform a substantially larger number of tests, which needs to be taken into account [13, 54]. As limited work has been done to investigate the threshold in different populations, there is currently no consensus on exactly what the modified threshold should be, and whether it should be the same for all populations. We have therefore used the standard threshold in the current study. It should be noted that revision of the threshold in the near future may move some of our significant signals to suggestive signals. For replication of previously detected signals, we selected a *p* threshold of 5 × 10^−3^, using an approach similar to that of Kuchenbaecker et al [40]. While this is less stringent compared with an LD-adjusted Bonferroni threshold, we observed that very few signals were replicated even at this relaxed threshold.

We acknowledge that our study was limited to FG, FI, HOMA-B and HOMA-IR. Including other phenotypes that were not measured in the AWI-Gen cohort, such as the glucose tolerance test, may provide more information on glycaemic traits as quantitative variables that indicate the risk of type 2 diabetes. Due to the lack of glycaemic trait GWASs in continental Africans, our main challenge in this study was obtaining a suitable replication dataset, given the unique genetic diversity of our study populations. It would have been ideal to use replication cohorts from continental African cohorts instead of African Americans or other global populations given the differences in environmental context and genetics. We recognise that the β (effect size) and MAF of the associated SNPs (Table 1) are close to or below that considered detectable based on our power calculations; hence this study is not adequately powered to detect signals with smaller effect size or lower MAFs. Additionally, the small sample size of the current analysis may have increased the likelihood of type II errors. Therefore, the results should be interpreted with these limitations in mind, and highlight the need for larger African cohorts to overcome the limitations.

A major strength of our study is that, to our knowledge, it is the largest GWAS of glycaemic traits in a continental African cohort, a population that has been historically under-studied in genomic research. Although the AWI-Gen sample size is still relatively small compared to cohorts with predominantly European, Asian and African American participants, this study serves as a starting point for further genomic studies of glycaemic traits. All study participants were recruited according to a standardised recruitment process, and the laboratory assays for FG and FI were performed in a single laboratory, thus limiting the potential for differences associated with the collection method and laboratory assays.

In summary, we identified novel associations with three glycaemic traits (FG, FI and HOMA-IR) in individuals from sub-Saharan Africa. The findings of this study add to the global catalogue of genetic associations with glycaemic traits. Further functional genomic analyses will be useful to shed light on the biological mechanisms underlying the role of these novel associations in glucose and insulin regulation and in type 2 diabetes pathogenesis. The results highlight the importance of broadening genetic research to include under-represented participants from continental Africa.

## Supplementary Information

Below is the link to the electronic supplementary material.

ESM1 (PDF 458 KB)

ESM2 (XLSX 726 KB)

## Data Availability

The dataset used in this study is available in the European Genome–Phenome Archive (EGA) database (https://ega-archive.org/) under study accession code EGAS00001002482. The phenotype dataset accession code is EGAD00001006425 and the genotype dataset accession code is EGAD00010001996. The availability of these datasets is subject to controlled access by the Data and Biospecimen Access Committee of the H3Africa Consortium. GWAS summary statistics are accessible through the NHGRI-EBI GWAS Catalog (https://www.ebi.ac.uk/gwas/; FG-BMIadjusted, GCST90503329; FG-BMIunadjusted, GCST90503330; FI-BMIadjusted, GCST90503331; FI-BMIunadjusted, GCST90503332; HOMA-B-BMIadjusted, GCST90503333; HOMA-B-BMIunadjusted, GCST90503334; HOMA-IR-BMIadjusted, GCST90503335; HOMA-IR-BMIunadjusted, GCST90503336). Other published datasets included in this study are referenced in the Methods section.

## Abbreviations

AWI-Gen: Africa Wits-INDEPTH Partnership for Genomics Studies
FG: Fasting glucose
FI: Fasting insulin
GWAS: Genome-wide association study
LD: Linkage disequilibrium
MAF: Minor allele frequency
QQ: Quantile–quantile

## Code availability

No custom codes were used in this study. We used publicly available software (referenced in the Methods section) in this research. The H3A-Africa GWAS pipeline was used for quality control analysis and fine mapping, and can be accessed at https://github.com/h3abionet/h3agwas. Other scripts used in this study are available at https://github.com/chirrie/GWAS-of-glycaemic-traits.
