# Supplementary material for: Genome-wide association study identifying novel risk variants associated with glycaemic traits in the continental African AWI-Gen cohort
Source: Diabetologia. 2025 Mar 1;68(6):1184–96. doi: 10.1007/s00125-025-06395-6 (PMC12069158; doi:10.1007/s00125-025-06395-6)
Supplement: Supplementary file 1 — ESM1 (PDF 458 KB) [file 125_2025_6395_MOESM1_ESM.pdf]

Genome-wide association study identifying novel risk variants associated with glycaemic traits in the continental African AWI-Gen cohort.

Electronic supplementary materials

ESM METHODS

Glycaemic trait measurements

A detailed description of the data and sample collection methods was presented by Ali et al. [1]. Briefly, fasting blood samples were obtained after an overnight fast by study participants. Fasting plasma glucose was assayed using a Randox Plus clinical chemistry analyser (UK) using a colorimetric method. The Randox glucose assay measurement range was 0.36–35 mmol/l. The coefficient of variation (CV) of the laboratory measurements for FG was less than 2.3%. Similarly, a solid-phase, enzyme-labelled chemiluminescent immunometric assay was used to determine fasting serum insulin concentrations using the Immulite 1000 chemistry analysis system (Siemens, Germany). The measuring range for the FI concentration was 2–300 uIU/ml, and its coefficient of variation (CV) was less than 2%. Homeostatic model assessment of insulin resistance (HOMA-IR) and homeostasis model assessment of beta-cell function (HOMA-B were calculated using previously reported formulas [2, 3].

Power calculation

Power calculations were performed using the gwas_power function in R (version 4.2) (https://github.com/kaustubhad/gwas-power/blob/master/power_calc_functions.R).This function calculates power for a range of beta and minor allele frequency values, for pre-determined sample size and *p* thresholds. We set the *p* threshold to genome-wide significance threshold (5×10^{-8}) and independently estimated the power for sample sizes corresponding to each trait (FG = 9,889, FI = 6,825, HOMA-IR = 6,500, and HOMA-B = 6,362).

SNP heritability and genetic correlations

The SNP heritability of the four glycaemic traits was determined using the restricted maximum likelihood method in GCTA-GREML version 1.93.253 [4]. For all analyses, we included age, sex, and the eight genetic principal components as covariates. Bivariate GCTA-GREML was used to investigate the genetic correlation between the four glycaemic traits (FG, FI, HOMA-IR, and HOMA-B). POPCORN program was used for trans-ancestry genetic correlation [5].

Genome-wide association analysis

Genome-wide association analyses were performed using the linear mixed model implemented in the BOLT-LMM software (v2.3.4) [6] for: FG, FI, HOMA-B, and HOMA-IR. Untransformed FG, log-transformed FI, log-transformed HOMA-B, and log-transformed HOMA-IR were used with sex, BMI, age, and eight principal components (PCs) fitted as covariates. As there is no consensus on the ideal *p* threshold for African GWAS, we employed the commonly used genome-wide significance criterion of $p<5 \times 10^{-8}$ [7]. Any SNPs with *p* values between 5×10^{-8} and 1×10^{-6} were considered to be suggestive. Manhattan and quantile-quantile

(QQ) plots were generated from GWAS results using the qqman package in R version 4.2.0 [8]. The locus zoom plots were generated using LocusZoom [9].

Functional Analysis of Associated Variants

The possible biological functions of genome-wide significant ($p<5 \times 10^{-8}$) variants were assessed using the Ensembl Variant Effect Predictor (VEP) [10]. The function of mapped genes was inferred using GeneCards [11]. Annotation and prioritization of genomic risk loci were performed using the Functional Mapping and Annotation (FUMA) web tool [12]. The African subset from the 1000 Genomes Project [13] was used as the reference data for linkage disequilibrium (LD) analyses. Gene-set and tissue expression analyses were performed using multi-marker analysis of genomic annotation (MAGMA, v1.6) in the FUMA toolkit. Gene-set analysis assessed the over-representation of biological functions based on gene ontology terms from the Molecular Signature Database (MsigDB v5.2) [14]. Gene sets with a $p<0.05$, corrected for multiple testing, were considered significantly enriched. Tissue expression analysis was performed using expression quantitative trait loci (eQTL) data from the Genotype Tissue Expression Project (GTEx v8) [15]. The significantly associated loci were cross-referenced with the GWAS Catalog in the NHGRI-EBI [16] to identify previously reported signals.

Fine mapping of associated loci

The program FINEMAP [17] implemented in the H3Agwas pipeline [18] was used to find candidate causal variants associated with the four glycaemic traits reported in this study. The full AWI-Gen genotype data and site-specific genotype data were used as LD references for fine mapping. FINEMAP employs a shotgun stochastic search technique (a Bayesian-based framework) to predict the posterior probabilities (pp) of causative variants using summary statistics and LD correlations among variants [17]. Credible sets of SNPs were created by ranking variants by their decreasing posterior probability (*pp*) to jointly account for 95% of the *pp* and were assumed to be driving the association. A log10 Bayes factor (log10BF) greater than two was used as a cut-off for selecting candidate causal variants following the recommendations of the FINEMAP authors (http://www.christianbenner.com/index_v1.1.html). Independently associated SNPs were selected using a stepwise model selection procedure in COJO-GCTA, using AWI-Gen genotype data as the LD reference data [19].

References

1. Ali SA, Soo C, Agongo G, et al (2018) Genomic and environmental risk factors for cardiometabolic diseases in Africa: methods used for Phase 1 of the AWI-Gen population cross-sectional study. Glob Health Action 11(sup2):1507133. https://doi.org/10.1080/16549716.2018.1507133

2. Bonora E, Formentini G, Calcaterra F, et al (2002) HOMA-Estimated Insulin Resistance Is an Independent Predictor of Cardiovascular Disease in Type 2 Diabetic Subjects: Prospective data from the Verona Diabetes Complications Study. Diabetes Care 25(7):1135–1141. https://doi.org/10.2337/diacare.25.7.1135

3. Matthews DR, Hosker JP, Rudenski AS, Naylor BA, Treacher DF, Turner RC (1985) Homeostasis model assessment: insulin resistance and β-cell function from fasting plasma glucose and insulin concentrations in man. Diabetologia 28(7):412–419. https://doi.org/10.1007/BF00280883

4. Yang J, Lee SH, Goddard ME, Visscher PM (2011) GCTA: a tool for genome-wide complex trait analysis. Am J Hum Genet 88(1):76–82. https://doi.org/10.1016/j.ajhg.2010.11.011

5. Brown BC, Ye CJ, Price AL, Zaitlen N (2016) Transethnic genetic-correlation estimates from summary statistics. Am J Hum Genet 99(1):76–88. https://doi.org/10.1016/j.ajhg.2016.05.001

6. Loh P-R, Tucker G, Bulik-Sullivan BK, et al (2015) Efficient Bayesian mixed-model analysis increases association power in large cohorts. Nat Genet 47(3):284–290. https://doi.org/10.1038/ng.3190

7. Choudhury A, Brandenburg J-T, Chikowore T, et al (2022) Meta-analysis of sub-Saharan African studies provides insights into genetic architecture of lipid traits. Nat Commun 13(1):2578. https://doi.org/10.1038/s41467-022-30098-w

8. Turner S (2018) qqman: an R package for visualizing GWAS results using QQ and manhattan plots. J. Open Source Softw. 3: 731. https://doi.org/10.21105/joss.00731

9. Boughton AP, Welch RP, Flickinger M, et al (2021) LocusZoom. js: Interactive and embeddable visualization of genetic association study results. Bioinformatics 37(18):3017–3018. https://doi.org/10.1093/bioinformatics/btab186

10. Martin FJ, Amode MR, Aneja A, et al (2023) Ensembl 2023. Nucleic Acids Res 51(D1):D933–D941. https://doi.org/10.1093/nar/gkac958

11. Safran M, Rosen N, Twik M, et al (2021) The GeneCards Suite. In: Abugessaisa I, Kasukawa T (eds) Practical Guide to Life Science Databases. Springer Nature Singapore, Singapore, pp 27–56

12. Watanabe K, Taskesen E, van Bochoven A, Posthuma D (2017) Functional mapping and annotation of

genetic associations with FUMA. Nat Commun 8(1):1826. https://doi.org/10.1038/s41467-017-01261-5

13. 1000 Genomes Project Consortium (2015) A global reference for human genetic variation. Nature 526(7571):68

14. Liberzon A, Birger C, Thorvaldsdóttir H, Ghandi M, Mesirov JP, Tamayo P (2015) The molecular signatures database hallmark gene set collection. Cell Syst 1(6):417–425. https://doi.org/10.1016/j.cels.2015.12.004

15. The GTEx Consortium, Aguet F, Anand S, et al (2020) The GTEx Consortium atlas of genetic regulatory effects across human tissues. Science 369(6509):1318–1330. https://doi.org/10.1126/science.aaz1776

16. Buniello A, MacArthur JAL, Cerezo M, et al (2019) The NHGRI-EBI GWAS Catalog of published genome-wide association studies, targeted arrays and summary statistics 2019. Nucleic Acids Res 47(D1):D1005–D1012. https://doi.org/10.1093/nar/gky1120

17. Benner C, Spencer CC, Havulinna AS, Salomaa V, Ripatti S, Pirinen M (2016) FINEMAP: efficient variable selection using summary data from genome-wide association studies. Bioinformatics 32(10):1493–1501. https://doi.org/10.1093/bioinformatics/btw018

18. Brandenburg J-T, Clark L, Botha G, et al (2022) H3AGWAS: a portable workflow for genome wide association studies. BMC Bioinformatics 23(1):498. https://doi.org/10.1186/s12859-022-05034-w

19. Yang J, Ferreira T, Morris AP, et al (2012) Conditional and joint multiple-SNP analysis of GWAS summary statistics identifies additional variants influencing complex traits. Nat Genet 44(4):369–375. https://doi.org/10.1038/ng.2213

ESM FIGURES

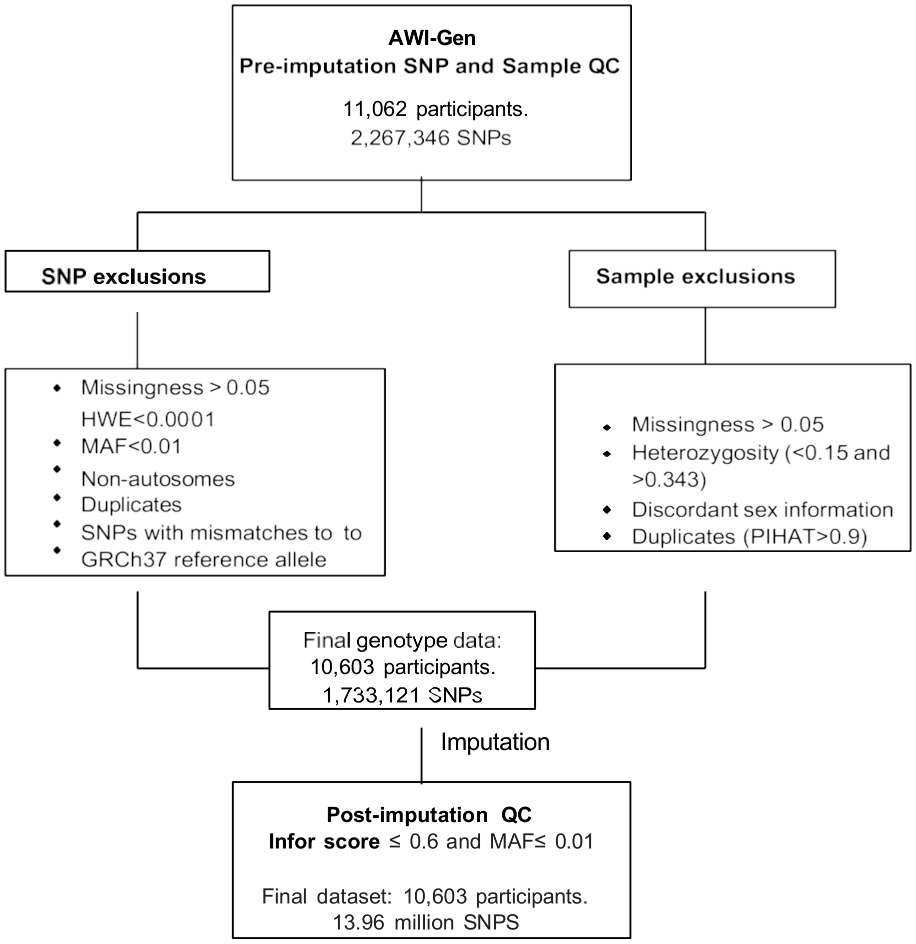

ESM Fig. 1: Flowchart showing the different quality control steps performed on AWI-Gen dataset

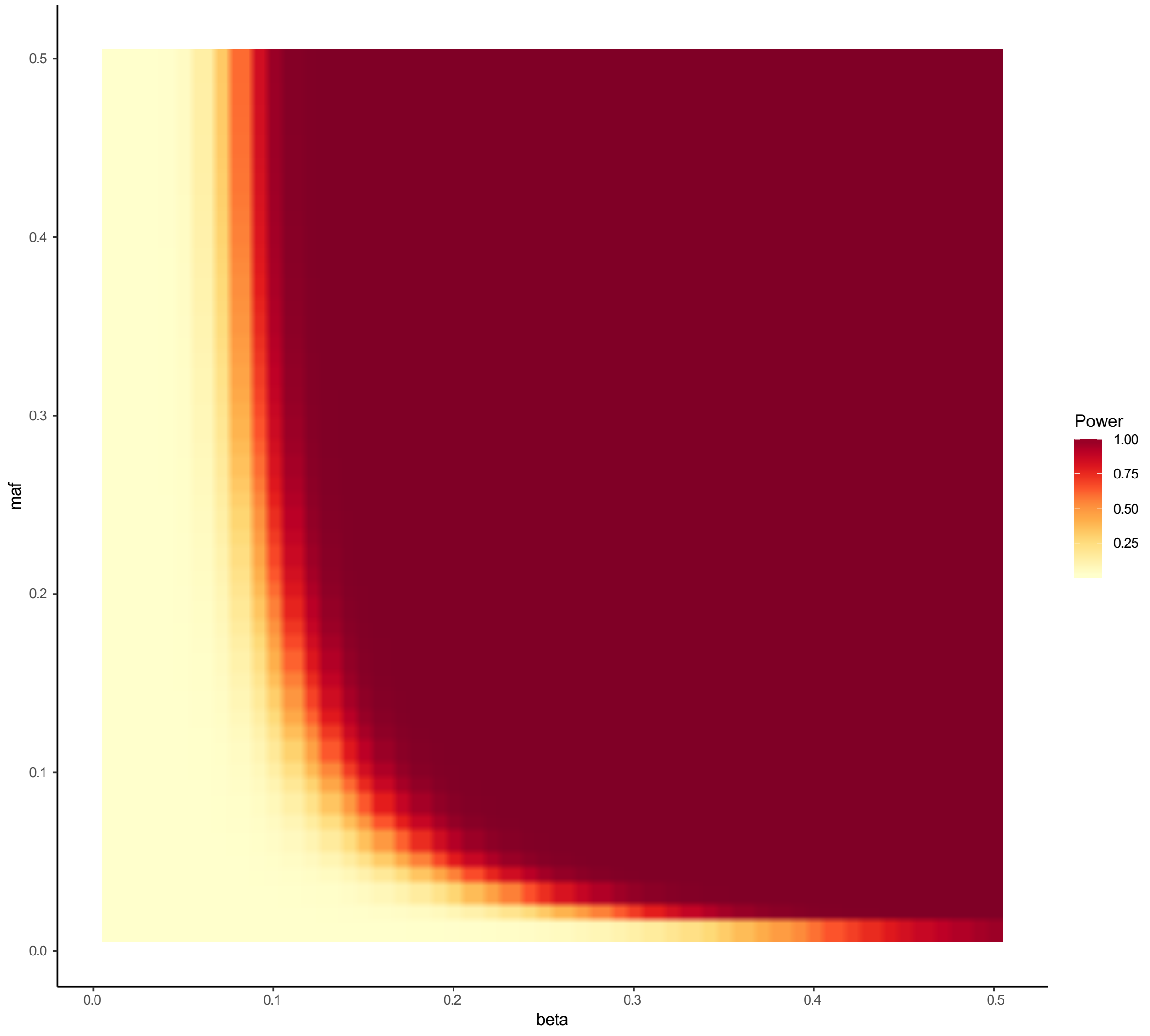

ESM Fig. 2: Power estimation for the GWAS of FG (as a quantitative trait) considering genetic effect β ranging from 0.01 to 0.5 and allele frequencies ranging from 0.01 to 0.5, with sample fixed to 9,889 and *p* = 5×10^{-8}). The x-axis represents beta values, whereas the y-axis represents the minor allele frequency values. The power values are represented by the colour gradient, with darker colour representing high power values, and light yellow representing low values. As beta increases, the power increases for any given value of MA

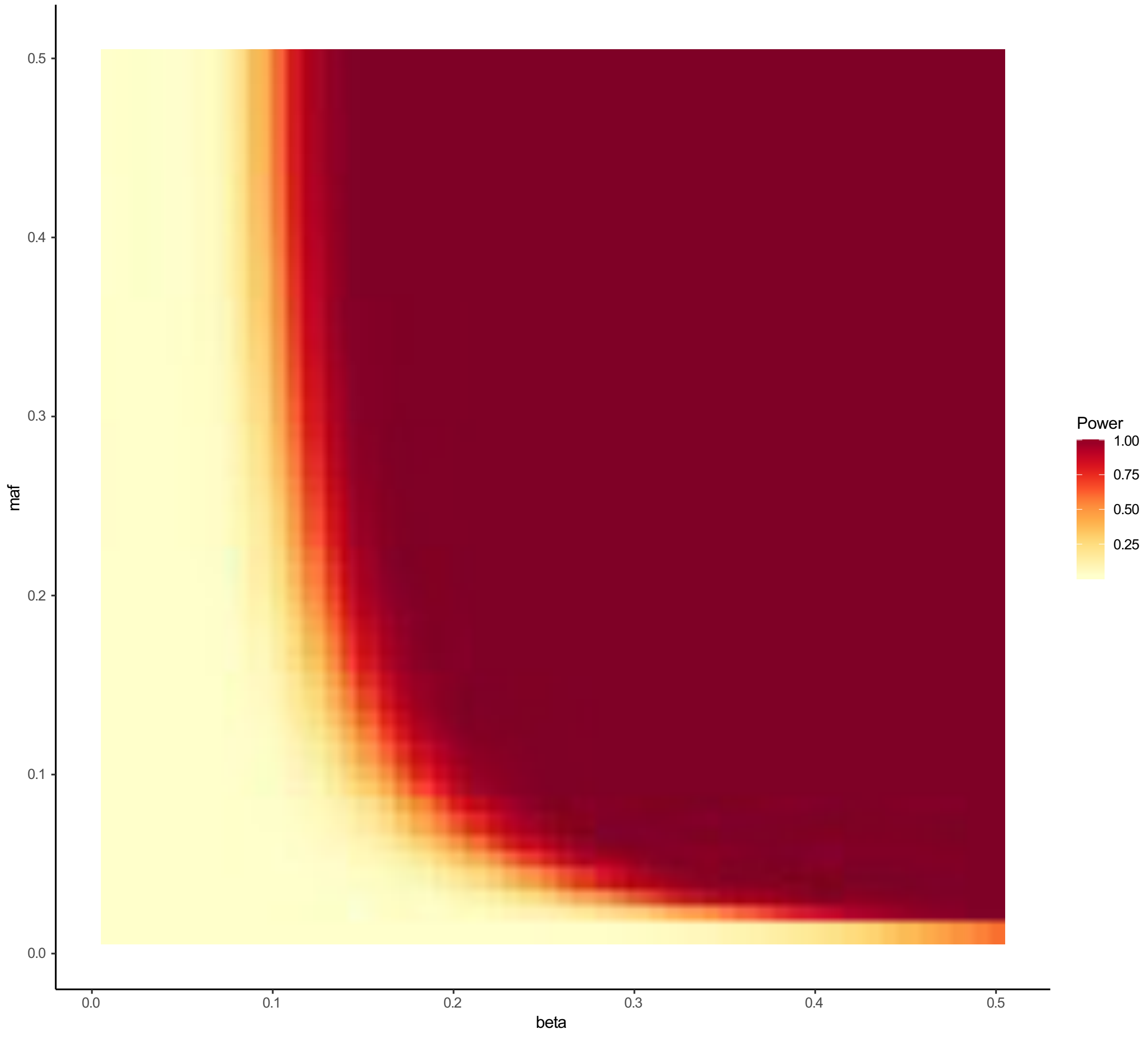

ESM Fig. 3: Power estimation for the GWAS of HOMA-B (as a quantitative trait) considering genetic effect β ranging from 0.01 to 0.5 and allele frequencies ranging from 0.01 to 0.5, with sample fixed to 6,362 and $p = 5 \times 10^{-8}$. The x-axis represents beta values, whereas y- represents minor allele frequency values

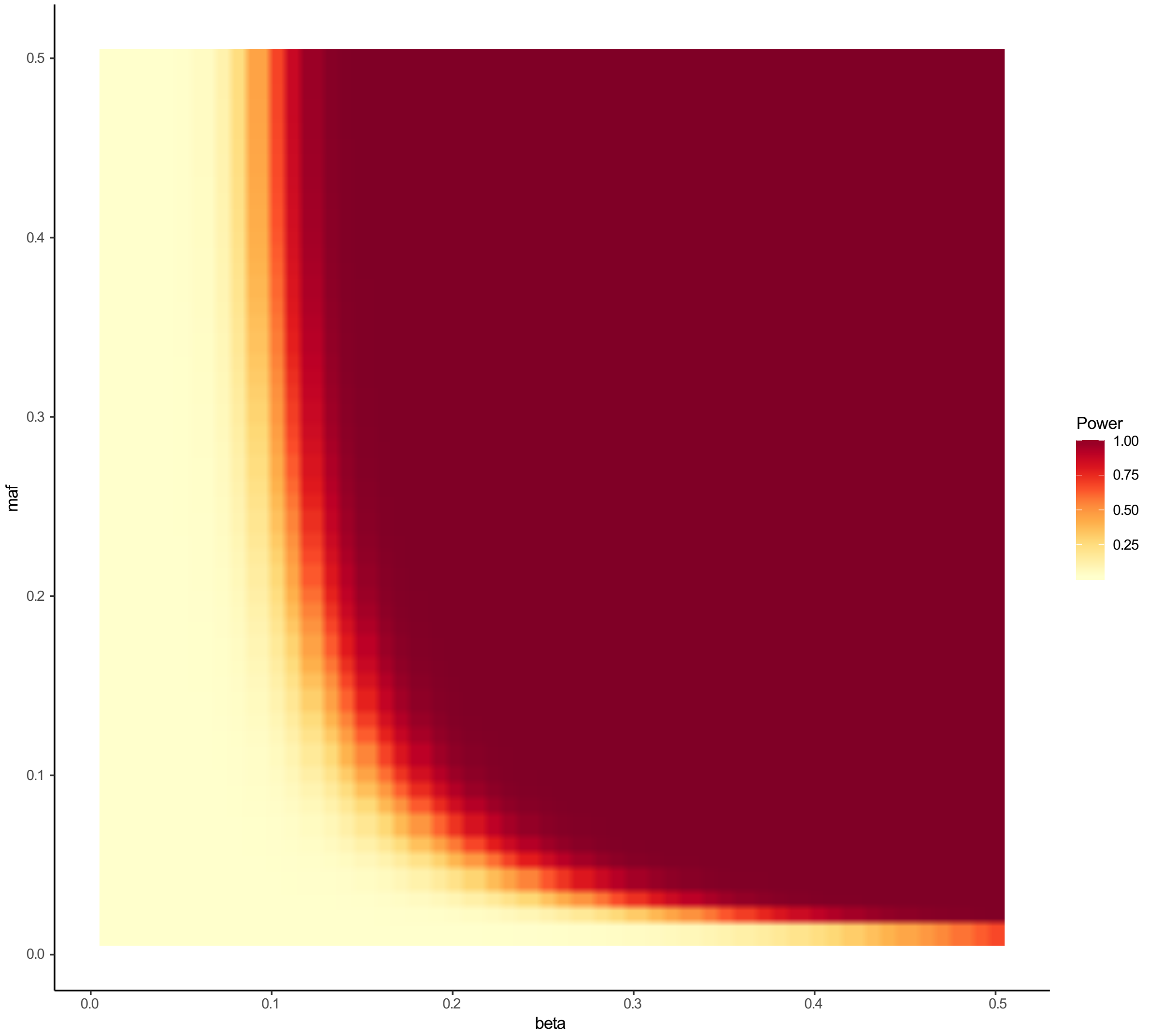

ESM Fig. 4: Power estimation for the GWAS of FI (as a quantitative trait) considering genetic effect β ranging from 0.01 to 0.5 and allele frequencies ranging from 0.01 to 0.05, with sample fixed to 6826 and p=5 × 10^{-8}). The x- axis represents beta values while y-axis represents minor allele frequency values

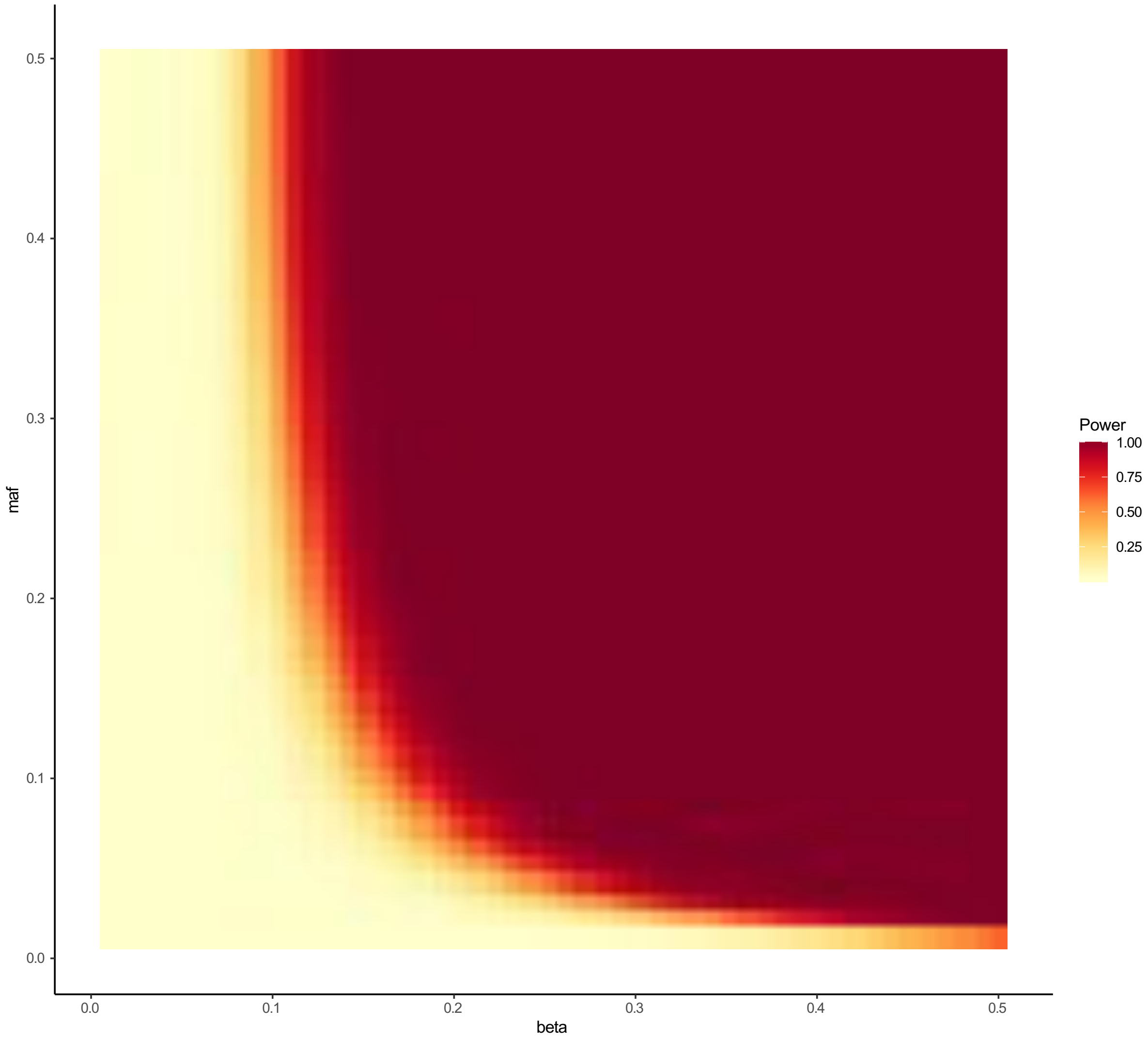

ESM Fig. 5: Power estimation for the GWAS of HOMA-IR (as a quantitative trait) considering genetic effect β ranging from 0.01 to 0.5 and allele frequencies ranging from 0.01 to 0.5, with sample fixed to 6,500 and p=5 × 10^{-8}). The x-axis represents beta values while the y-axis represents minor allele frequency values

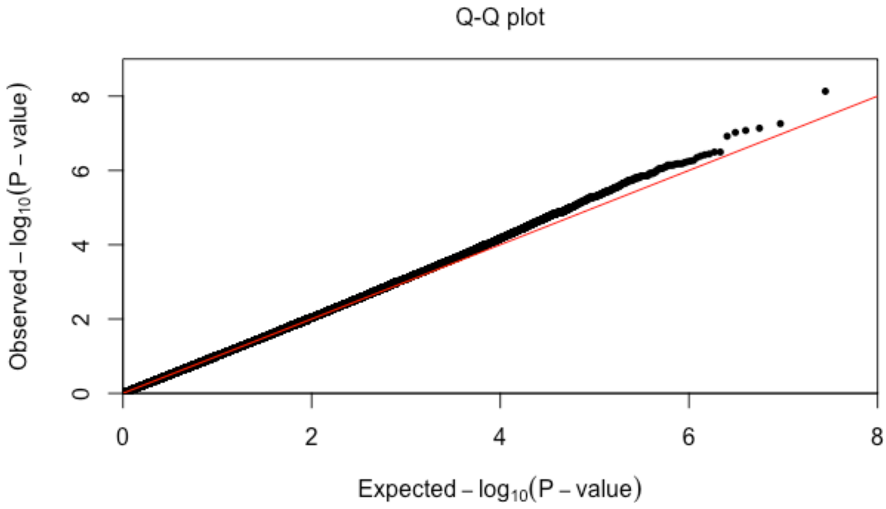

ESM Fig. 6: Quantile-quantile plot of *p* values for fasting glucose trait in continental African individuals in AWI-Gen dataset.

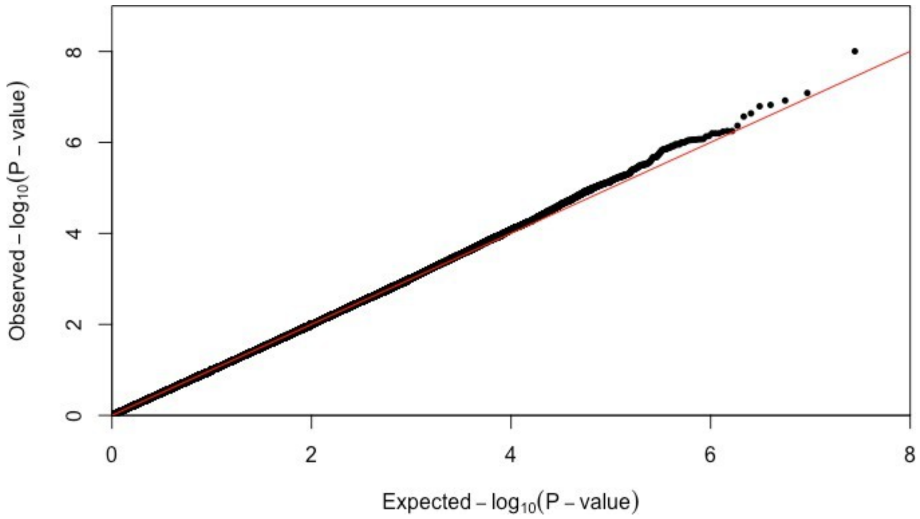

ESM Fig. 7. Quantile-quantile plot of *p* values for fasting insulin trait in continental African individuals in AWI-Gen dataset

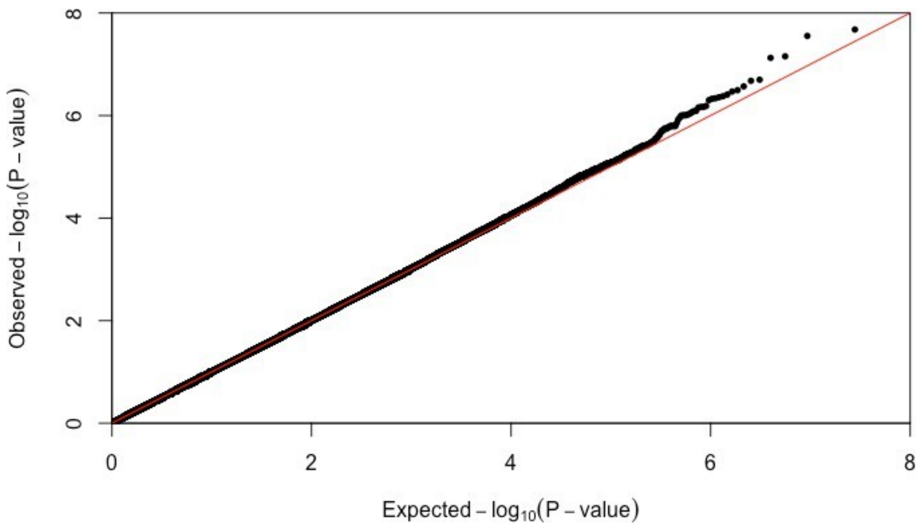

ESM Fig. 8. Quantile-quantile plot of *p* values for HOMA-IR trait in continental African individuals in the AWI-Gen dataset.

ESM Tables (see the separate file)

ESM Table 1: Descriptive statistics for AWI-Gen cohorts

ESM Table 2: Loci that reached suggestive significance ($p<1 \times 10^{-6}$) for glycemic traits in AWI-Gen cohort

ESM Table 3: Genome-wide significant locus reported in MAGIC European datasets reported to be associated with fasting glucose that replicated in AWI-Gen cohort ($p<0.005$)

ESM Table 4: Genome-wide significant locus reported in European population in UKBB reported to be associated with fasting glucose that replicated in AWI-Gen cohort ($p<0.005$)

ESM Table 5: Genome-wide significant locus reported in transethnic study of random glucose in 476,326 individuals that replicated in AWI-Gen cohort ($p<0.005$)

ESM Table 6: Finemapping of FG, FI and HOMA-IR lead variants in AWI-Gen cohort

ESM Table 7: Genome-wide association signals for fasting insulin and HOMA-IR reported previously that were found in AWI-Gen cohort.
